# Rhabdomyolysis Associated with Severe Levodopa-Induced Dyskinesia in Parkinson’s Disease: A Report of Two Cases and Literature Review

**DOI:** 10.5334/tohm.641

**Published:** 2021-09-30

**Authors:** Yuvadee Pitakpatapee, Jindapa Srikajon, Tanita Sangpeamsook, Prachaya Srivanitchapoom

**Affiliations:** 1 Division of Neurology, Department of Medicine, Faculty of Medicine Siriraj Hospital, Mahidol University, 2 Wanglang Road, Bangkok-noi, Bangkok 10700, Thailand

**Keywords:** Dyskinesia, Dyskinesia hyperpyrexia, Hyperkinetic emergencies, Parkinson’s disease

## Abstract

**Background:**

Rhabdomyolysis associated with levodopa-induced dyskinesia (Rhab-LID) is an extremely rare, life-threatening, but treatable condition in patients with Parkinson’s disease (PD).

**Case report:**

We reported two cases of Rhab-LID. The first case was a 64-year-old man presenting with severe generalized dyskinesia with elevated serum creatine kinase (CK) level. He was diagnosed with Rhab-LID owing to unpredictable gastric emptying time. The second case was a 61-year-old woman presenting with fever, myalgia, and disabling dyskinesia with elevated serum CK. She was diagnosed with dyskinesia-hyperpyrexia syndrome (DHS) due to increasing dosage of ropinirole and infection. Dopaminergic medications were stopped, and supportive care was initiated in both cases with excellent outcomes.

**Conclusion:**

Early recognition, stopping dopaminergic medications, treating precipitating causes, and proper supportive treatment can provide favorable outcomes.

## Introduction

Levodopa-induced dyskinesia (LID) is a common motor complication in patients with Parkinson’s disease (PD). It usually develops in the mid- to advanced stage of PD. Prevalence of LID is approximately 30% and 50% after 5-year and 10-year of initiating levodopa therapy, respectively.

The common phenomenology of dyskinesia in PD is chorea; however, it may present as a combination of chorea, dystonia, and athetosis [1]. Usually, dyskinesia occurs on the side of the body that was firstly affected by motor symptoms. However, it can also manifest in other parts of the body such as facial muscles, tongue, neck, and trunk [2]. Peak-dose dyskinesia occurring at the time of peak plasma level of levodopa is the most common type of dyskinesia, following by off-period dystonia which usually involving legs before taking the next dose of levodopa. The least common type is diphasic dyskinesia which starts after 10–15 minutes after taking levodopa, and dyskinesia re-emerges when the plasma level of levodopa decrease [1,2]. Potential risk factors for developing LID are younger age at onset of diagnosis of PD, exposure to high-dose of levodopa (especially higher than 400–600 mg/day), female sex, low body weight, and akinetic-rigid subtype of PD [2]. LID may contribute towards various negative consequences in PD patients including both the physical and psychological domains. LID may cause falls, self-injury, exhaustion, fatigue, social embarrassment, anxiety, depression, and physical dependence.

Rhabdomyolysis associated with levodopa-induced dyskinesia is an extremely rare condition in advanced PD [3]. It can be a life-threatening condition named dyskinesia-hyperpyrexia syndrome, which is characterized by high serum creatine kinase (CK) level, acute kidney injury (AKI), fever, myalgia, and altered consciousness [4,5]. Potential complications of rhabdomyolysis according to this syndrome are acute kidney injury and electrolyte imbalance, e.g., hyperkalemia, which leads to cardiac arrhythmia and death in the early stage. Moreover, disseminated intravascular coagulation could be found as a late serious complication [6]. However, early detection and prompt treatment could lead to a favorable prognosis. Herein, we report two patients with Rhab-LID who received early diagnosis and management, resulting in excellent clinical outcomes.

## Case Report

### Case 1

A 64-year-old man with a 10-year history of PD presented with severe generalized dyskinesia for two days. He had developed both diphasic dyskinesia and wearing-off in the past two years. He had a history of worsening abdominal distension and constipation over the past several years. He had been taking levodopa/benserazide (LB) 650 mg/day, entacapone 500 mg/day, piribedil 150 mg/day, and benzhexol 2 mg/day without recent dosage adjustment. He denied history of statin use, trauma, and infection. On admission, he had severe generalized dyskinesia with profuse sweating. His body temperature was 99.5 °F. His consciousness was good. Laboratory findings showed no leukocytosis, elevated blood urea nitrogen (BUN) (37.9 mg/dL, reference value 7–20 mg/dL), elevated serum creatinine (1.26 mg/dL, reference value 0.5–1.5 mg/dL), and elevated serum CK (4246 U/L, reference value 20–195 U/L). Urinalysis showed no myoglobinuria. Rhab-LID with acute kidney injury (AKI) was diagnosed. Intravenous fluid replacement and intravenous diazepam were promptly administered. All anti-parkinsonian medications were stopped. His dyskinesia markedly improved three days later, and serum CK normalized within five days. RYR1 gene mutation test was negative. He was discharged after six days of hospitalization on a regimen of LB 950 mg/day only.

### Case 2

A 61-year-old woman with a 10-year history of PD presented with severe disabling dyskinesia and myalgia for four days. She had had troublesome diphasic dyskinesia within the past six years. She had been taking LB 875 mg/day and entacapone 800 mg/day. Ropinirole extended-release had been recently added and up-titrated to 4 mg/day within the past three weeks. She denied a history of current statin use and trauma. On admission, she was alert but mildly confused. Her body temperature was 100.2°F. She had severe generalized dyskinesia. Laboratory findings were leukocytosis (white blood cell count 10,290/uL), elevated BUN (37.8 mg/dL), and elevated serum creatinine (1.27 mg/dL), and highly elevated serum CK level (12,094 U/L). Urinalysis showed evidence of urinary tract infection (UTI) with myoglobinuria. Dyskinesia-hyperpyrexia syndrome (DHS) with AKI was diagnosed. Intravenous fluids replacement and empirical antibiotics were immediately administered. All anti-parkinsonian medications were stopped, and oral clonazepam was started. Her symptoms and consciousness were improved within four days. Autoimmune myositis antibody panels were negative. She was discharged after six days of hospitalization with a serum CK of 633 U/L. Her anti-parkinsonian medication was adjusted to LB 500 mg/day only.

## Discussion

Rhab-LID may be an under-reported and potentially life-threatening complication in LID patients. We reviewed the 15 cases of Rhab-LID and DHS reported in the literature, including the present cases (***Table 1***) [3,4,5,7,8,9,10,11,12,13,1,2,3,4,5,6,7,8,9,10].

**Table 1:** Clinical features of the two cases in the present report and previously reported cases.


REFERENCE	AGE (YEARS) /SEX	PD DURATION (YEARS)	MEDICATIONS (MG/DAY)	DURATION OF DYSKINESIA BEFORE ADMISSION (DAYS)	BODY TEMPERATURE (^O^F)	SIGNS AND SYMPTOMS	LEVEL OF SERUM CREATINE KINASE (IU/L)	DIAGNOSIS	POSSIBLE TRIGGERS	MANAGEMENT	OUTCOME

Factor and Molho, 2000	50/M	6	L/C 600/60 Adding pramipexole with up-titration	< 1	N/A	Generalized dyskinesia, shortness of breath, diaphoresis, and marked dehydration	> 21,000	Rhab-LID without AKI	Adding pramipexole	Stopped all medications, IV fluid replacement	Improved

Gil-Navarro and Grandas, 2010	68/F	12	L/C/E 750/250/1,000, pramipexole 4, amantadine 200	2	106.2	Generalized dyskinesia, drowsiness, fever, tachycardia, visual and auditory hallucination	1,455	DHS without AKI	N/A	Stopped pramipexole, adding quetiapine 25 mg/day, IV fluid replacement	Improved and D/C 7 days after admission

Lyoo and Lee, 2011	74/M	17	L/C 3,000/300, L/B CR 400/100	9	100.8	Generalized dyskinesia, mild rigidity, good consciousness	24,651	DHS with AKI	Increasing dose of levodopa	Stopped all antiparkinsonian medications, IV midazolam 0.4-0.8 ug/min/kg	Improved and D/C 9 days after admission

Bektas et al., 2014	76/F	15	L/C/E 1500/375/2,000	3	Reported normal BT	Generalized dyskinesia, good consciousness	2,253	Rhab-LID with AKI	Increasing dose of levodopa	Hemodialysis, lowered levodopa dosage	Died due to severe pneumonia with sepsis

Taguchi et al., 2015	70/F	13	L 600, pramipexole IR 3 then switched to ER 3, selegiline 5	7	104.5	Generalized dyskinesia, fever, tachycardia, visual hallucination	>30,000	DHS with AKI	Switching pramipexole IR to ER formulation	Tapered down of all antiparkinsonian medications	Improved

Herreros-Rodriguez and Sánchez-Ferro, 2016	76/F	18	LED 670.5	N/A	104.4	Dyskinesia, fever, good consciousness	257	DHS without AKI	High environmental temperature	Switched to LCIG	Improved

Sánchez-Herrera et al., 2016	66/F	16	LCIG L=1,450, safinamide 100, amantadine 200, ropinirole 8	8	104.4	Generalized dyskinesia, fever, confusion, visual hallucination	7,177	DHS without AKI	Adding ropinirole, high environmental temperature	Stopped all antiparkinsonian medications, IM clorazepate 50 mg, IV diazepam 10 mg, IV midazolam 10 mg	Improved

Baek et al., 2017	1^st^ visit; 74/F	23	L 375, amantadine 200, pramipexole ER 1.075	1	104.5	Generalized dyskinesia, fever, confusion, visual hallucination	1,023	DHS with AKI	Fracture of ribs	Stopped pramipexole ER, and amantadine, IV midazolam	Improved and D/C 6 days after admission

	2^nd^ visit; 75/F	24	L 500, amantadine 200, pramipexole ER 1.075	2	100.8	Generalized dyskinesia, fever, confusion, visual hallucination	661	DHS with AKI	Fall with trauma to the left flank	Stopped pramipexole ER, reduced levodopa to 300 mg/day, IV fluid replacement	Improved and D/C 9 days after admission

Sarchioto et al., 2018	Case 1; 80/M	17	LCIG L=1500, amantadine 200, pramipexole 1, sertraline 50	N/A	107.6	Generalized dyskinesia, fever, confusion, lethargy	16,040	DHS with AKI	Cholecystitis, high environmental temperature	IV fluid replacement, IV ATB, stopped pramipexole, and amantadine, reduced LCIG to 700 mg/day	Died due to multi-organ failure 5 days after admission

	Case 2; 76/F	18	LCIG L=1,200, pramiplexole 1, clozapine 25, venlafaxine 75, zolpidem 10	< 1	105.8	Generalized dyskinesia, stupor, tachycardia, respiratory distress, dehydration	2,967	DHS	Infection, high environmental temperature	N/A	Died within 1 day after admission

	Case 3; 79/F	30	LCIG L=1,250	4	103.1	Generalized dyskinesia, fever, dehydration	1,967	DHS with AKI	Infection, high environmental temperature	IV fluid replacement, IV ATB, reduced LCIG to 675 mg/day	Improved and D/C 6 days after admission

Novelli et al., 2019	62/M	34	STN-DBS, L/C 2,000/200, E 1,600	3 hours	105.3	Generalized dyskinesia, fever, tachycardia, confusion	4,891	DHS	Urinary tract infection, high environmental temperature	IV fluid replacement, IV ATB, Reduced setting of DBS, reduced L/C to 750/75, and E to 1,200	Improved and D/C 4 days after admission

Cases from the present report	Case 1; 64/M	10	L 650, E 500, piribedil 150, benzhexol 2	2	99.5	Generalized dyskinesia, sweating, good consciousness	4,246	Rhabdomyolysis Induced LID	Delayed gastric emptying time	Stopped all medications, IV fluid replacement, intravenous diazepam	Improved and D/C 6 days after admission

	Case 2; 61/F	10	L 875, E 800, ropinirole 4	4	100.2	Generalized dyskinesia, fever, dehydration, myalgia, good consciousness	12,094	DHS with AKI	Urinary tract infection, increasing dose of ropinirole	Stopped all medications, IV fluid replacement, intravenous diazepam	Improved and D/C 5 days after admission


D/C, discharge; ER, extennded release; F, female; IR, immediate release; IV, intravenous; LCIG, levodopa-carbidopa intestinal gel; M, male; NA, not available; PD, Parkinson’s disease.

The exact pathophysiology of Rhab-LID and DHS is not well understood. All cases had long durations of PD with motor complications and had taken high dosages of dopaminergic medications (levodopa equivalent dose > 600 mg/day). Generalized dyskinesia was shown in all cases, and 4 of 15 cases also demonstrated autonomic instabilities such as diaphoresis, tachycardia, and tachypnea. Alteration of consciousness, for example, stupor, confusion, and visual hallucination, were detected in around 50% of cases. Our second case showed mild confusion, but the first case had good consciousness. Fortunately, our cases did not show any psychiatric manifestations. An incremental dopaminergic cell loss due to disease progression may play a major role in developing Rhab-LID in LID [7]. Other potential precipitating factors reported in the reviewed cases were up-titration of dopaminergic medications, switching dopaminergic medication from immediate-release to extended-release formulation, infection, trauma, dehydration, and living in high environmental temperature [5,7,8,9,10,11,12,13]. Increasing the dosage of dopaminergic drugs was the most commonly associated factor (5 of 15 cases). Two possible mechanisms related to this factor are increment of pulsatile presynaptic dopamine-releasing coupling with reduced postsynaptic buffering capacity of dopaminergic receptors [13] and alteration of striatal synaptic plasticity, resulting in an increment of long-term potentiation and absence of depotentiation. Dehydration, physical stress, psychological stress, increased body temperature due to infection, living in a high ambient temperature, and autonomic dysfunction may increase dopamine release and increase the sensitivity of dopaminergic receptors [4]. Six of the 15 cases were associated with a combination of two precipitating factors.

In our first case, the potential precipitating factor may have been unpredictable gastrointestinal dysmotility, which may have increased the bioavailability of levodopa due to unpredictable levodopa absorption [7]. The second case could have been precipitated by increasing the dosage of ropinirole and UTI.

Twelve of the 15 incidences had a favorable outcome. The keys to success for managing patients with Rhab-LID include early recognition, prompt reduction in dopaminergic medications, treating possible precipitating factors, and proper supportive treatment, such as rehydration or prescribing antipyretics.

In conclusion, physicians should consider the possibility of Rhab-LID as a complication in patients with advanced PD presenting with severe generalized dyskinesia regardless of whether any precipitating factor can be identified. Early recognition and proper management can provide favorable clinical outcomes along with minimization of morbidity and mortality rates.
